# Immune Response to BNT162b2 in Solid Organ Transplant Recipients: Negative Impact of Mycophenolate and High Responsiveness of SARS-CoV-2 Recovered Subjects against Delta Variant

**DOI:** 10.3390/microorganisms9122622

**Published:** 2021-12-18

**Authors:** Irene Cassaniti, Federica Bergami, Francesca Arena, Jose Camilla Sammartino, Alessandro Ferrari, Federica Zavaglio, Irene Curti, Elena Percivalle, Federica Meloni, Laura Pandolfi, Carlo Pellegrini, Annalisa Turco, Elena Seminari, Eleonora Francesca Pattonieri, Marilena Gregorini, Teresa Rampino, Antonella Sarasini, Daniele Lilleri, Fausto Baldanti

**Affiliations:** 1 Molecular Virology Unit, Microbiology and Virology Department, Fondazione IRCCS Policlinico San Matteo, 27100 Pavia, Italy; i.cassaniti@smatteo.pv.it (I.C.); federica.bergami01@universitadipavia.it (F.B.); arena.franci@gmail.com (F.A.); jose.sammartino@iusspavia.it (J.C.S.); alessandro.ferrari@smatteo.pv.it (A.F.); fede.zavaglio90@gmail.com (F.Z.); irene.curti01@universitadipavia.it (I.C.); e.percivalle@smatteo.pv.it (E.P.); a.sarasini@smatteo.pv.it (A.S.); f.baldanti@smatteo.pv.it (F.B.); 2 Transplant Center Unit, Fondazione IRCCS Policlinico San Matteo, 27100 Pavia, Italy; f.meloni@smatteo.pv.it; 3 Research Laboratory of Lung Diseases, Section of Cell Biology, Fondazione IRCCS Policlinico San Matteo, 27100 Pavia, Italy; l.pandolfi@smatteo.pv.it; 4 Cardiac Surgery, Department of Intensive Medicine, Fondazione IRCCS Policlinico San Matteo, 27100 Pavia, Italy; c.pellegrini@smatteo.pv.it (C.P.); a.turco@smatteo.pv.it (A.T.); 5 Department of Clinical, Surgical, Diagnostic and Pediatric Sciences, University of Pavia, 27100 Pavia, Italy; 6 Infectious Disease Department, Fondazione IRCCS Policlinico San Matteo, 27100 Pavia, Italy; e.seminari@smatteo.pv.it; 7 Unit of Nephrology, Dialysis and Transplantation, Fondazione IRCCS Policlinico San Matteo, 27100 Pavia, Italy; e.pattonieri@smatteo.pv.it (E.F.P.); m.gregorini@smatteo.pv.it (M.G.); t.rampino@smatteo.pv.it (T.R.)

**Keywords:** solid organ transplant recipients, BNT162b2 vaccine, SARS-CoV-2, immune response

## Abstract

The immunogenicity of severe acute respiratory syndrome 2 virus (SARS-CoV-2) vaccines in immunocompromised patients remains to be further explored. Here, we evaluated the immunogenicity elicited by complete vaccination with BNT162b2 vaccine in solid organ transplant recipients (SOTRs). A cohort of 110 SOTRs from Northern Italy were vaccinated with two doses of BNT162b2 mRNA vaccine and prospectively monitored at baseline and after 42 days. Both SARS-CoV-2 naïve and recovered subjects were included. Humoral response elicited by vaccination, including SARS-CoV-2 neutralizing antibodies (SARS-CoV-2 NT Abs), was evaluated; additionally, ex-vivo ELISpot assay was performed for the quantification of Spike-specific T-cell response. Results were compared with those obtained in a cohort of healthy subjects. In a subset of patients, humoral and T-cell responses against delta variant were also evaluated. Less than 20% of transplanted subjects developed a positive humoral and cell-mediated response after complete vaccination schedule. Overall, median levels of immune response elicited by vaccination were significantly lower with respect to controls in SARS-CoV-2 naïve transplant, but not in SARS-CoV-2 recovered transplanted patients. Additionally, a significant impairment of both humoral and cell-mediated response was observed in mycophenolate-treated patients. Positive delta-SARS-CoV-2 NT Abs levels were detected in almost all the SARS-CoV-2 recovered subjects but not in previously uninfected patients. Our study supports previous observations of a low level of seroconversion after vaccination in transplanted patients.

## 1. Introduction

Coronavirus disease 2019 (COVID-19) caused by severe acute respiratory syndrome virus 2 (SARS-CoV-2) has severely impacted solid organ transplant recipients (SOTRs), not only for a substantial decrease of transplant care practice but also for an increased risk of morbidity and mortality linked to SARS-CoV-2 infection [1]. It has been reported that COVID-19 is associated with a mortality rate of 28% and hospitalization rate of 78% in SOTRs [2]. Similarly, European studies confirmed a mortality rate ranging from 19% to 30% in SOTRs [3,4,5,6]. Thus, prophylactic strategies are mandatory in order to avoid SARS-CoV-2 infection in SOTRs. So far, immunogenicity and efficacy of SARS-CoV-2 mRNA vaccines, including BNT162b2, has been widely documented in immunocompetent subjects [7,8] including those subjects with previous SARS-CoV-2 exposure [9,10].

Similarly, the potential immunogenicity and efficacy of the SARS-CoV-2 vaccines in immunocompromised patients has been intensively studied. The impaired humoral response in solid organ transplant recipients (SOTRs) has been documented [11,12]. In the first 6 months after transplantation, patients receive the highest level of immunosuppression and it is recommended to avoid vaccinations because of a likely lack of response. After 6 months, vaccination monitoring-guided reduction of immunosuppression can improve antiviral immunity [13] and response to vaccination. Thus, an impairment of immune response after vaccination might be hypothesized in SOTRs, as well as a reduced protection elicited by vaccine [14,15]. So far, the protective antibody level as well as the potential role of cellular immune response in protection against SARS-CoV-2 infection remain to be elucidated.

The main aim of the present study was to evaluate the immunogenicity of mRNA SARS-CoV-2 vaccine in SOTRs receiving immunosuppressive therapy. In particular, it is conceivable that different immunosuppressive drugs might differently affect immune response elicited by vaccination. In our prospective longitudinal study, we studied SOTRs vaccinated with BNT162b2 vaccine, analyzing both humoral and cell-mediated responses.

## 2. Materials and Methods

### 2.1. Patient Enrolment

A total of 110 SOTRs (66 males and 44 females; median age 49, range 23–82) were enrolled at time of SARS-CoV-2 vaccination with BNT162b2 vaccine and samples were collected the same day of first dose administration (T0) and three weeks after complete vaccination (T2). Serum samples were collected for evaluation of SARS-CoV-2 total and neutralizing antibodies while heparinized whole blood samples were used for peripheral mononuclear cells (PBMC) isolation and quantification of Spike-specific T-cell response. All the patients were enrolled at Fondazione IRCCS Policlinico San Matteo, according to Helsinki declaration and after approval of local ethical committee “Comitato Etico Pavia” (P-20210000232) on 10 February 2021.

Characteristics of enrolled patients, including type of transplant and immunosuppressive therapy, are shown in Table 1. Retrospectively, 74 SARS-CoV-2 seronegative and 8 SARS-CoV-2 recovered healthcare workers with no comorbidities (22 males and 60 females; median age 46, range 26–69) were included as a healthy control group.

### 2.2. Humoral Response Elicited by BNT162b2 Vaccine

Chemiluminescent assay (Liaison SARS-CoV-2 trimeric, Diasorin, Saluggia, Italy) was used according to manufacturers’ instructions using serum samples. Results higher than 33.8 BAU/mL were given as positive. In order to exclude asymptomatic infection during the overall period of follow-up, anti-N response was determined using the chemiluminescent assay Elecsys Anti-SARS-CoV-2 S (Roche Diagnostics, Rotkreuz, Switzerland) at T2.

SARS-CovV-2 neutralizing antibody (NT Abs) titre was determined as previously reported [16]. Briefly, 50 µL of serum in serial fourfold dilution was added in two wells of a flat bottom tissue culture microtiter plate (COSTAR, Corning Incorporated, Corning, NY, USA). The same volume of 100 TCID50 of SARS-CoV-2 strain (including PV10734 European-derived strain, alpha and delta variants) was added and plates were incubated at 33 °C in 5% CO_2_, according to our local protocol [17]. After 1 h incubation at 33 °C 5% CO_2_, VERO E6 cells were added to each well. After 72 h of incubation at the same conditions, plates were stained with Gram’s crystal violet solution (Merck KGaA, Damstadt, Germany) plus 5% formaldehyde 40% m/v (Carlo ErbaSpA, Arese, Italy) for 30 min. Microtiter plates were then washed under running water. Wells were scored to evaluate the degree of cytopathic effect (CPE) compared to the virus control. Blue staining of wells indicated the presence of neutralizing antibodies. Neutralizing titer was the maximum dilution with the reduction of 90% of CPE. All the experiments were performed in BSL3 facility. Results higher or equal to 1:10 serum titer were considered positive, according to our protocol [16].

### 2.3. SARS-CoV-2 T Cell Response Elicited by BNT162b2 Vaccine

Briefly, PBMC (2 × 10^5^/ 100 μL culture medium per well) were stimulated in duplicate for 24 h in 96-well plates (previously coated with anti-IFN-γ monoclonal capture antibody) with peptide pools (15 mers, overlapping by 10 aminoacids, Pepscan, Lelystad, The Netherlands) representative of the spike protein (S) at the final concentration of 0.25 µg/mL. Phytoheamagglutinin (PHA; 5 µg/mL) was used as positive control, and medium alone as negative control. In a subset of samples, viral lysate of PV10734 European-derived strain and delta variants were used as antigens for PBMC stimulation, in order to assess the T-cell response against the most relevant VOC. Enzyme linked immunospot assay was performed according to our previous protocol [18]. The net spots per million PBMC was calculated by subtracting the number of spots in response to negative control from the number of spots in response to the S antigen. Responses ≥10 net spots/million PBMC were considered positive based on background results obtained with negative control (mean SFC + 2SD).

### 2.4. Statistical Analysis

Frequency and percentage of subjects positive for serological assay, SARS-CoV-2 NT Abs test and Spike-specific T-cell response were determined and comparison between groups was made by Fisher’s exact test. Quantitative data were given as median and interquartile range (IQR) and comparisons were performed by Mann–Whitney U test (two groups) or Kruskal–Wallis with Dunn’s post hoc test (three or more groups). Correlation between quantitative variables was calculated using Spearman test. A multiple linear regression analysis was adopted to identify independent predictors of immune response to the vaccine. Parameters significantly associated with immune response in univariate analysis were included in the multiple regression models. Immune parameters were log-transformed for the analysis. All the assays were two-tailed and *p* value < 0.05 was considered significant. GraphPad Prism 8.3.0 (GraphPad Software Inc., La Jolla, CA, USA) was used for all the analyses.

## 3. Results

### 3.1. Humoral and Cell-Mediated Response Elicited by mRNA BNT162b2 Was Suboptimal in SARS-CoV-2 Naïve Transplanted Patients

**Figure 1:**
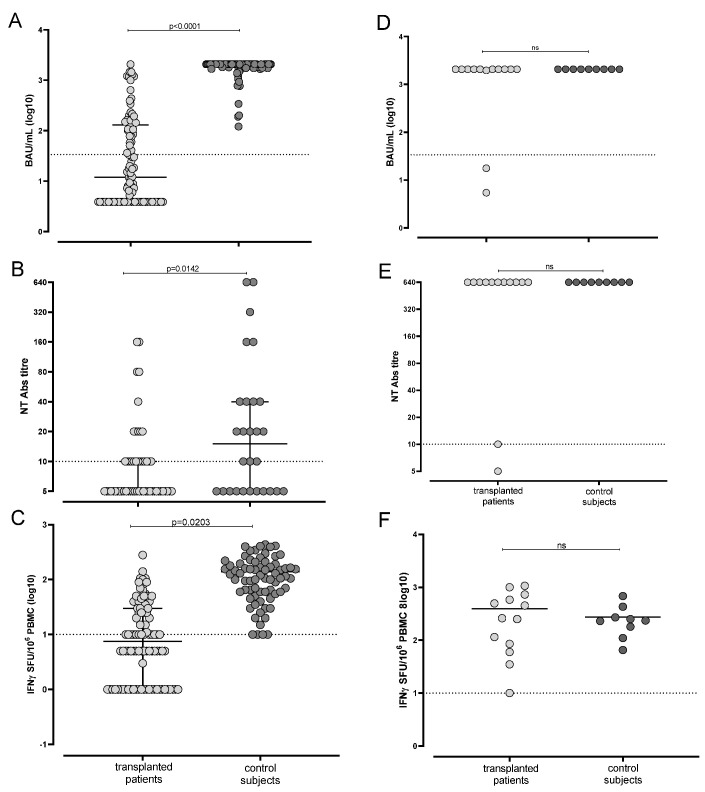
Total IgG SARS-CoV-2 response measured by Trimeric assay (**A**), SARS-CoV-2 NT Abs level (**B**) and Spike-specific response-cell response (**C**) were compared in SARS-CoV-2 naïve BNT162b2 vaccinated transplanted patients (*n* = 97) and healthy controls (*n* = 74). Total IgG SARS-CoV-2 response measured by Trimeric assay (**D**), SARS-CoV-2 NT Abs level (**E**) and Spike-specific response-cell response (**F**) were compared in SARS-CoV-2 recovered BNT162b2 vaccinated transplanted patients (*n* = 13) and healthy controls (*n* = 9). *p* values were obtained by Mann–Whitney test and given for each graph.

Out of 110 enrolled subjects, 97 (88.2%) SOTRs were SARS-CoV-2 seronegative at baseline and had not experienced a previous SARS-CoV-2 infection. Of them, 36 (37.1%) showed a positive result for Trimeric IgG assay at T2 and median level was 12 (IQR 3.9–131.6) BAU/mL. As control, all the immunocompetent healthcare workers reached a positive level of Trimeric Spike response (median ≥ 2080 [IQR 1746– ≥2080] BAU/mL) (Figure 1A). On the other hand, 46/97 (47.4%) SOTRs were positive for SARS-CoV-2 NT Abs at T2 (overall median 1:5 IQR 1:5–1:20) while all the healthcare workers were positive for SARS-CoV-2 NT Abs at T2 showing a median response of 1:320 (1:320–≥1:640; Figure 1B). In terms of cell-mediated response against spike antigen, only 49/97 (50.5%) showed a positive response after two vaccine doses (median 10 [IQR 0–30] IFN-γ SFU/10^6^ PBMC) while 73/74 healthcare workers were positive for Spike-specific T-cell response at T2 (median 110.5 [IQR 56.3–187.5] IFN-γ SFU/10^6^ PBMC; Figure 1C). Overall, only 17/97 (17.5%) patients were considered “full responders” after vaccination.

Of note, 13/110 (11.8%) SOTRs were previously infected with SARS-CoV-2 at baseline, since SARS-CoV-2 IgG and/or NT Abs were detected as positive. All these subjects reported sustained positive levels of IgG at T2 (median ≥ 2080 [IQR 2018–≥2080] BAU/mL in SOTRs and ≥2080 BAU/mL in all immunocompetent healthcare workers; *p* = 0.4857) (Figure 1D). Looking at SARS-CoV-2 NT Abs in Figure 1E, the overall median was ≥1:640 in 11/13 transplanted patients and in all healthy controls (*p* = 0.4935). Finally, all of the 13 SARS-CoV-2 seropositive patients developed a positive Spike-specific T-cell response (median 72.5 [IQR 5–260] IFN-γ SFU/10^6^ PBMC) that was not statistically different from median Spike-specific T-cell response observed in healthy controls (median 235 [IQR 145–350] IFN-γ SFU/10^6^ PBMC; *p* = 0.6589) (Figure 1F). Negative anti-N IgG was detected at T2 in all but one SARS-CoV-2 naïve subjects, suggesting that only one patient experienced a SARS-CoV-2 asymptomatic infection during the follow-up period.

### 3.2. Immune Response Elicited by Vaccination in Transplanted Patients Is Associated with Age and Time after Transplant

The role of age and years after transplant in SARS-CoV-2 immune response elicited by vaccination was analyzed. A weak negative correlation was observed between age and serological result (r = −0.3; *p* = 0.0031 for Trimeric assay), but also between age and NT Abs level (r = −0.23; *p* = 0.0207) as well as between age and S-ELISpot response (r = −0.25; *p* = 0.0148). Conversely, correlation with age was not observed in healthy controls.

On the other hand, no correlation between years after transplant and SARS-CoV-2 immune response was observed. However, since the most intensive immunosuppression normally occurs during the first year after transplant, we separately analyzed SARS-CoV-2 immune response in 12/97 SARS-CoV-2 naïve subjects vaccinated within one year after transplantation and 77/97 patients vaccinated later after transplantation. Both SARS-CoV-2 NT Abs level and S-ELISpot response were not significantly different between the two groups, while a significant difference was observed for IgG response (median 3.9 [IQR 3.9–10.7] BAU/mL and 17.9 [IQR 3.9–151.6] BAU/mL; *p* = 0.0127). No association between sex and immune response was observed (data not shown).

### 3.3. Reduced Humoral and Cell-Mediated Immune Response to BNT162b2 in Mycophenolate-Treated Patients

**Figure 2:**
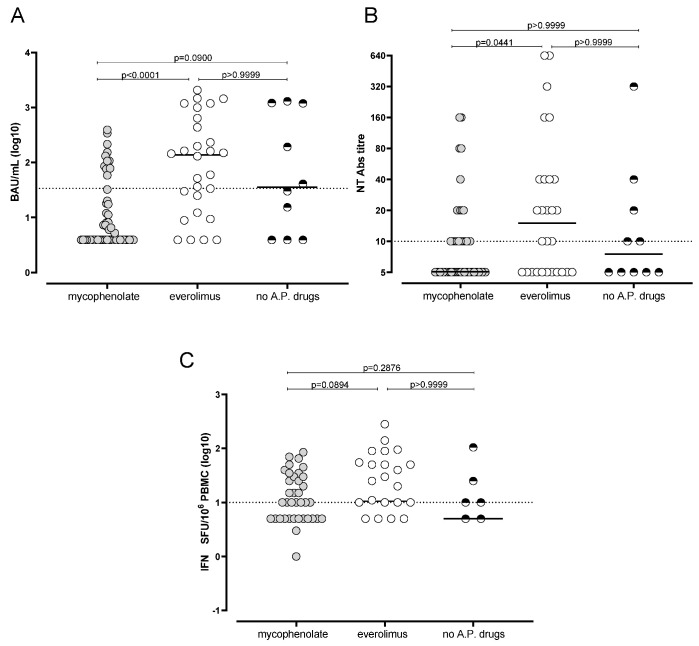
Total IgG SARS-CoV-2 response measured by Trimeric (**A**) assay, SARS-CoV-2 NT Abs level (**B**) and Spike-specific response-cell response (**C**) were compared in subjects treated with mycophenolate (*n* = 55), everolimus (*n* = 28) or without antiproliferative drugs (*n* = 10). *p* values were obtained by Kruskall–Wallis test and given for each graph. No A.P. drugs: no antiproliferative drug administered.

Based on immunosuppressive regimens, subjects were divided in three groups: (i) SOTRs treated with mycophenolate (55/97; 56.7%), (ii) SOTRs treated with everolimus (28/97; 28.9%) and (iii) SOTRs (10/97; 10.3%) without anti-proliferative treatment. In order to avoid confounding factors, the four SOTRs (4.1%) treated with a combination of everolimus and mycophenolate were excluded from the analysis. As shown in Figure 2, the overall immune response elicited by mRNA BNT162b2 vaccine was significantly reduced in subjects treated with mycophenolate. In detail, the median level of total Spike-specific IgG was 3.9 IQR 3.9–23 BAU/mL in the mycophenolate group while medians of 137.3 IQR 15.4–588.9 BAU/mL and 35.4 IQR 3.9–1199 BAU/mL were observed in the everolimus group and in those subjects without antiproliferative drugs, respectively (Figure 2A). The overall rate of positive subjects in the mycophenolate group (13/55; 23.6%) was significantly lower with respect to the everolimus group (18/28; 64.3%; *p* = 0.0006). The difference was not statistically significant when rate of positive subjects was compared between the mycophenolate group and the no anti-proliferative drug groups (5/10; *p* = 0.1240). Similarly, no differences were also reported between everolimus and no anti-proliferative drugs (*p* = 0.4726).

Median level of SARS-CoV-2 NT Abs was 1:5 (IQR 1:5–1:10) in the mycophenolate group, 1:14 (1:5–1:40) in everolimus group and 1:7 (IQR 1:5–1:25) in no anti-proliferative drugs group (Figure 2B). Additionally, the rate of positive SOTRs at T2 for SARS-CoV-2 NT Abs was not statistically different between the three groups. In detail, the rate was 23/55 (41.8%) and 17/28 (60.7%) in mycophenolate and everolimus groups (*p* = 0.1122) and 5/10 (50%) in the group of patients with no anti-proliferative drugs administration (*p* = 0.7341 and 0.4726, respectively).

Finally, Spike-specific T-cell response measured by ELISpot assay was analysed in patients receiving the different immunosuppressive drugs. SOTRs treated with mycophenolate showed a median Spike-specific T-cell response of 5.0 (0.0–16.3) IFN-γ SFU/10^6^ PBMC while those treated with everolimus had a median Spike-specific T-cell response of 10.5 (5–50) IFN-γ SFU/10^6^ PBMC and 5.0 (0.0–13.8) IFN-γ SFU/10^6^ PBMC in the group of SOTRs with no anti-proliferative drugs administration (Figure 2C). Despite the rate of subjects positive for Spike-specific T-cell response being lower in the mycophenolate group (24/55; 43.6%) with respect to everolimus (18/28; 64.3%) and no anti-proliferative drugs 6/10 (60%) groups, the difference was not statistically significant (*p* = 0.2367 and *p* = 0.4931). Similarly, no differences were observed between everolimus and no anti-proliferative drug groups in terms of rate of Spike-specific T-cell response positive subjects (*p* > 0.9999).

No differences in terms of SARS-CoV-2 specific response were observed between SOTRs treated with tacrolimus or cyclosporine. Similarly, the levels of steroids did not affect immune response elicited by vaccination. Finally, type of transplanted organ did not influence vaccine response (data not shown).

In a multivariate linear regression model including age, time after transplant and use of mycophenolate, we found that the use of mycophenolate was independently associated with a lower IgG antibody level and to a lower NT titer. Association between time after transplant and IgG antibody level was significant with the IgG S Trimeric assay. Age and time after transplant were not independently associated with NT titer, and no factor appeared independently associated with T-cell response (Table 2).

### 3.4. Humoral and T-Cell-Mediated Immune Response against SARS-CoV-2 Variants Were More Efficient in Previously Infected Subjects

**Figure 3:**
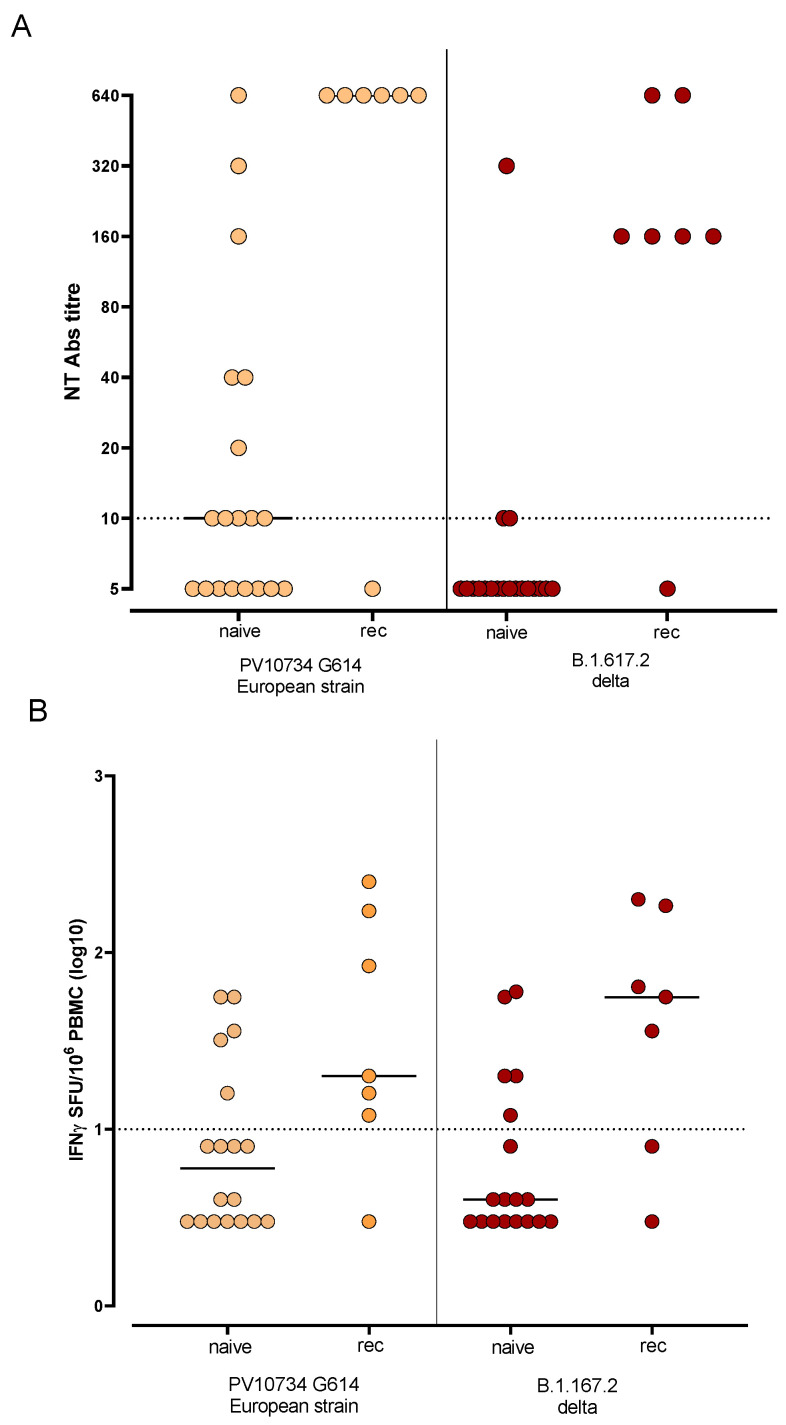
SARS-CoV-2 NT Abs (**A**) and T-cell response (**B**) against reference SARS-CoV-2 strain (PV10734 G614 European strain) and delta variant (B.1.167.2) were tested in 19 naïve and 7 recovered (rec) SOTRs. Median level is shown for each response.

SARS-CoV-2 NT Abs against delta variant was tested in 26 subjects, including 7 SARS-CoV-2 recovered subjects. The same subjects were also tested for SARS-CoV-2 T-cell response against delta variant. Overall, 11/19 (57.9%) naïve vaccinated subjects were positive for SARS-CoV-2 NT Abs against SARS-CoV-2 reference strain while only 3/19 (15.8%) showed a positive NT Abs level against delta variant (*p* = 0.0170). Of note, no difference was observed in SARS-CoV-2 recovered subjects (Figure 3A). On the other hand, 5/19 (26.3%) naïve SOTRs were positive for T-cell response against both the reference strain and delta variant, while 5/7 (71.4%) and 6/7 (85.7%) recovered SARS-CoV-2 subjects were positive for T-cell response against the reference strain and delta variant, respectively (Figure 3B).

## 4. Discussion

In this study, we provide an analysis of humoral and cell-mediated response elicited by mRNA BNT162b2 vaccination in a cohort of 110 SOTRs from Northern Italy. As major result, we report that BNT162b2 vaccinated transplanted patients with no history of previous SARS-CoV-2 showed a suboptimal response to vaccination three weeks after complete schedule SOTRs showed a seroconversion, according to previous results [19,20]. Looking at only serological response, about 30% of patients showed a positive anti-Spike IgG response, while 47% and 50% of transplanted patients were positive for SARS-CoV-2 NT Abs and Spike-specific T-cell response, respectively. Similarly, looking at the overall IgG response, an immunization rate lower than 50% was observed in transplanted patients after two doses of mRNA-1273 vaccine [21]. Our results reported higher level of immunization in comparison to a French cohort [22] with only 4% of positive transplanted patients after complete BNT162b2 vaccination.

We observed a higher level of immunization in terms of cell-mediated response with respect to humoral response, as observed by others [23]. Similarly, as previously reported by Bertrand and colleagues, the rate of kidney transplant recipients who were positive for Spike-specific T-cell response was about 60% [24]. This finding raises the question if, even in absence of positive antibody level, a sustained T-cell response might be related to protection against COVID-19. Additionally, the role of potential cross-reactive T-cell response derived from other common human coronavirus infections should be considered.

So far, cell-mediated response represents a valuable tool for the evaluation of vaccine immunogenicity in immunocompromised patients, since the only serological approach might underestimate the rate of responder subjects [25,26]. Of note, no correlation was found between time after vaccination and vaccine-related immune response.

The lower rate of positive response to vaccination may represent a real concern since the risk of SARS-CoV-2 infection might be higher with respect to general population. So far, the administration of a third dose of vaccine might be necessary in immunosuppressed patients. In this setting, it has been recently observed that humoral and cell-mediated responses elicited by a third dose of the BNT162b2 vaccine in primary non-responder SOTRs is similar to that observed in de novo responders after two doses of vaccines [27,28].

Additionally, we observed that BNT162b2 vaccinated transplanted patients who were previously exposed to SARS-CoV-2 infection showed a sustained response, both humoral and cell-mediated, as previously reported [29], suggesting that hybrid immunization (viral infection followed by mRNA vaccine) led to an increase in vaccine immunogenicity.

As expected, we observed that humoral and cell-mediated response elicited by vaccination was reduced in subjects transplanted less than 18 months ago, and in subjects treated with mycophenolate, which may have an effect on impairing post-vaccine responses [30], especially in terms of humoral response. Similarly, humoral response to influenza vaccination is impaired in subjects treated with mycophenolate [31]. Conversely, no difference in terms of SARS-CoV-2 immune response was observed when different CNIs were used. This result was in contrast to what has been previously reported by others, who observed an association between Tacrolimus treatment and weak response to vaccination [25]. A weak inverse correlation between age and vaccine response was also observed in transplant recipients, but not in healthy control subjects.

Other factors have been independently correlated with rate of response elicited by vaccination, including corticosteroid treatment and type of mRNA vaccination [32,33].

It is known that the delta variant is able to induce immune escape and neutralization titer reduction of the vaccine immune response against Delta variant is higher than that observed for alpha variant [34,35].

Thus, as previously observed in healthcare workers and other transplanted patient cohorts [35,36], SARS-CoV-2 NT Abs level against delta variant was observed in SARS-CoV-2 recovered subjects but not in naïve subjects, suggesting that the hybrid immunization might also improve the overall response against the delta variant that is now widely spread in our country. On the other hand, SARS-CoV-2 T-cell response against delta variant was similar to that observed against the reference strain, not only for SARS-CoV-2 recovered but also for SARS-CoV-2 naïve SOTRs, suggesting that conserved epitopes might stimulate a T-cell response against different strains.

Looking at the humoral response, the use of NT Abs should be suggested in combination with serological assays since it augments the detection rate of subjects with positive antibodies.

As a major limitation, this is a monocentric study that includes only kidney and thoracic transplanted patients. No data are available on different types of solid organ transplants and only a low number of SARS-CoV-2 recovered SOTRs was considered.

## 5. Conclusions

In conclusion, BNT162b2 vaccination does not provide a detectable immune response in 70% of SOTRs, especially in case of mycophenolate treatment. On the other hand, triple antigen exposure appears to elicit an immune response similar to that observed in immunocompetent subjects. Further analyses on larger sample settings are required and prospective studies evaluating the long-term protection in immunocompromised patients are mandatory.

## Figures and Tables

**Table 1:** Demographic and clinical characteristics of enrolled patients.

	Number (%)
Male gender	66 (60%)
Type of transplant	
Heart	22 (20%)
Lung	26 (23.6%)
Kidney	62 (56.4%)
CNIs	
Cyclosporine	22 (20%)
Tacrolimus	83 (75.5%)
No	5 * (4.5%)
Anti-proliferative drug	
Mycophenolate	62 (56.4%)
Everolimus	30 (27.3%)
Mycophenolate and everolimus	6 (5.4%)
No	12 (10.9%)
Steroid level	
Low doses (<5mg/day)	68 (61.8%)
High doses (>5 mg/day)	3 (2.7%)
No	39 (35.5%)
Time after transplant	
Less than 1 year	12 (10.9%)
Between 1 and 5 years	45 (40.9%)
More than 5 years	53 (48.2%)
SARS-CoV-2 positivity at T0	
No	97 (88.2%)
Yes	13 (11.8%)

Legend CNIs: calcineurin inhibitors; T0: baseline time point.* Three patients received sirolimus in place of CNIs.

**Table 2:** Multiple linear regression analysis of factors potentially associated with vaccine response in transplant recipients.

Dependent Variable	Independent Variable	Estimate β Coefficient	95% Confidence Interval	*p* Value
S Trimeric (Log_10_BAU/mL)	Intercept	2.690	1.903 to 3.468	<0.001
	Age	−0.014	−0.028 to 0.000	0.054
	Time after transplant <18 months	−0.561	−1.030 to −0.091	0.020
	Use of mycophenolate	−0.806	−1.110 to −0.498	<0.001
Nt Abs (Log_10_ titer)	Intercept	1.480	0.930 to 2.030	<0.001
	Age	−0.00437	−0.014 to 0.005	0.380
	Time after transplant <18 months	−0.218	−0.547 to 0.111	0.192
	Use of mycophenolate	−0.264	−0.480 to −0.048	0.017
Spike-specific T cells (Log_10_ Spots)	Intercept	1.280	0.530 to 2.020	0.001
	Age	−0.006	−0.019 to 0.0078	0.407
	Time after transplant <18 months	−0.033	−0.475 to 0.408	0.881
	Use of mycophenolate	−0.200	−0.489 to 0.090	0.174

## Data Availability

Data available on request due to restrictions (privacy and ethical).
